# 慢性淋巴细胞白血病中BIM诱导伊布替尼耐药机制研究

**DOI:** 10.3760/cma.j.cn121090-20241121-00466

**Published:** 2025-02

**Authors:** 加乐 张, 必慧 潘, 佳竹 吴, 祎琳 孔, 莉 王, 卫 徐

**Affiliations:** 南京医科大学第一附属医院（江苏省人民医院）血液科，南京 210029 Department of Hematology, The First Affiliated Hospital of Nanjing Medical University（Jiangsu Province Hospital）, Nanjing 21009, China

**Keywords:** 白血病，淋巴细胞，慢性，B细胞, 伊布替尼, 耐药, BIM, 自噬, Leukemia, lymphocytic, chronic, B-cell, Ibrutinib, Drug resistant, BIM, Autophagy

## Abstract

**目的:**

探究BCL2家族蛋白BIM在慢性淋巴细胞白血病（CLL）伊布替尼耐药中的作用，分析其对凋亡和自噬的调控机制。

**方法:**

通过RNA测序检测CLL患者样本、CLL细胞株（MEC1细胞株）和耐药细胞株（MR）中BCL2家族表达变化。蛋白印迹法分析药物敏感细胞株凋亡时BIM蛋白表达变化，并用shRNA敲低BIM探讨其对增殖和凋亡的影响。利用RNA测序和自噬抑制剂氯喹观察MR自噬水平变化。

**结果:**

CLL原代细胞及MEC1细胞株耐药前后，BIM表达显著下调（*P*<0.0001）。在CLL细胞中敲低BIM可抑制由伊布替尼诱导的细胞凋亡（均*P*<0.05）。此外，在MR中可以观察到保护性自噬水平的提高，通过氯喹及小干扰RNA抑制自噬反应后可促进细胞凋亡。BIM敲低细胞株中LC3-Ⅱ蛋白表达增加（*P*<0.01），提示BIM降低可介导自噬激活。

**结论:**

BIM低表达是CLL中伊布替尼耐药的重要因素，其通过激活保护性自噬促进耐药，这为改善CLL治疗提供了新靶点。

## Abstract (en)

**Objective:**

To investigate the relationship between the BCL2 family protein BIM and ibrutinib resistance in chronic lymphocytic leukemia（CLL）and to analyze its regulatory mechanisms on apoptosis and autophagy.

**Methods:**

RNA sequencing（RNA-seq）was used to examine changes in the expression of BCL2 family proteins in samples from patients with CLL, MEC1 cell lines, and ibrutinib-resistant cell lines（MR）. Western blot was used to analyze changes in BIM protein expression during apoptosis in MR. shRNA knockdown was used to assess the effects of BIM on cell proliferation and apoptosis. RNA-seq and the autophagy inhibitor chloroquine treatment were used to study autophagy-related changes in MR.

**Results:**

BIM expression was significantly downregulated before and after drug resistance in CLL primary cells and MEC1 cell lines（*P*<0.0001）. Knockdown of BIM in CLL cells inhibited ibrutinib-induced apoptosis and promoted cell proliferation（*P*<0.05 for both）. In addition, protective autophagy was increased in MR and apoptosis was increased after administration of chloroquine and small interfering RNA. The increased expression of LC3-Ⅱ protein in BIM-knockdown cell lines（*P*<0.01）suggested that reduction of BIM may mediate autophagy activation.

**Conclusion:**

Downregulation of BIM may be a key factor in promoting ibrutinib resistance in CLL by activating protective autophagy. These findings provided a potential target for improving CLL treatment.

慢性淋巴细胞白血病（CLL）是一种常见的成人白血病，在西方国家占成人白血病的40％[1]。布鲁顿酪氨酸激酶（BTK）抑制剂伊布替尼可阻断BCR信号通路，抑制CLL细胞增殖并诱导凋亡[2]。尽管伊布替尼疗效显著，但耐药问题日益突出[3]。目前已知BTK和PLCG2位点突变与耐药相关，但仍有20％～30％的患者在耐药时未能检测到相应突变[4]，因此仍需探索新的耐药机制。线粒体凋亡是程序性死亡的重要途径，由BCL2家族蛋白调控。BCL2家族包括促凋亡蛋白（如BAX和BAK）、抗凋亡蛋白（如BCL2、MCL-1）及BH3-only蛋白（如BIM、BAD），BCL2家族蛋白的异常表达会导致抗癌药物耐药[5]。研究发现，CLL中MCL-1/BAX、BCL2/BAX的比值与治疗相关[6]，而BIM表达减少会削弱药物促凋亡作用，诱导继发性耐药[7]。

自噬是细胞的一种特殊死亡方式，通过形成自噬体并与溶酶体融合维持细胞稳态[8]。自噬既能清除异常细胞以抑制肿瘤，也能提高肿瘤细胞生存能力。CLL患者自噬基因表达升高，提示自噬活跃状态[9]。在抗癌治疗中，自噬通过削弱药物的凋亡作用诱导耐药[10]。伊布替尼治疗后CLL细胞自噬水平上升[11]，其在耐药机制中的作用值得探索。本研究旨在探讨CLL伊布替尼耐药前后BCL2家族蛋白的表达变化，通过研究BIM对凋亡和自噬的调控，探索自噬在耐药中的作用，为克服伊布替尼耐药提供新策略。

## 材料与方法

1. CLL原代细胞及CLL细胞株：获得患者知情同意后，在江苏省人民医院收集3例伊布替尼耐药且无BTK和PLCG2位点突变的CLL患者（男2例、女1例，年龄分别为55、62、76岁，IGHV均有突变，无TP53缺失或突变）的外周血标本。分别在服用伊布替尼前的基线期和服用伊布替尼后出现疾病进展的2个时间点收集匹配的对照标本。患者标本外周血单个核细胞（PBMC）细胞分离后，使用CD19磁珠（美天旎生物技术有限公司，德国）分选出的CD19^+^ B淋巴细胞。本研究CLL细胞株实验均使用MEC1细胞株。

2. 实时定量聚合酶链式反应（qRT-PCR）：收集细胞后根据RNA抽提试剂盒（上海奕杉生物科技有限公司，中国上海）说明书步骤提取RNA，参照逆转录试剂盒（南京诺唯赞生物科技股份有限公司，中国江苏）配比，使用PCR仪逆转录得到cDNA。cDNA可放置在−20 °C冰箱长期保存。qPCR测定目的基因表达，内参为GAPDH，记录样本CT值，计算3个复孔间平均CT值。根据CT值计算目的基因相对表达量＝2^−ΔΔCT^。引物序列（生工生物工程上海有限公司，中国上海）见表1。

**表1:** 实时定量聚合酶链式反应引物序列

引物名称	引物序列
BIM-F	AGACAGAGCCACAAGGTATTTT
BIM-R	GTATCTCGGCTCCGCAAAGA
GAPDH-F	GACAGTCAGCCGCATCTTCT
GAPDH-R	AAATGAGCCCCAGCCTTCTC

**注** F：正向；R：反向

3. 蛋白质印迹法（Western blot）：收集细胞，离心后弃上清，根据计数细胞量加入含蛋白酶和磷酸酶抑制剂（Bimake生物科技有限公司，美国）的RIPA溶液（北京索莱宝科技公司）裂解细胞。经SDS-PAGE电泳转移至聚偏二氟乙烯膜（PVDF膜），5％脱脂奶粉封闭2 h。TBST洗涤后，一抗4 °C孵育过夜、二抗室温孵育90 min。利用凝胶成像仪显影，并对条带灰度值进行半定量统计。使用抗体有：BIM、LC3、P62、Beclin-1、PUMA、PARP、Cleaved PARP抗体（Cell Signaling Technology公司，美国）；BCL-xL、MCL-1、BCL2、BAX抗体（Abcam公司，美国）；GAPDH抗体（Proteintech group公司，美国）

4. 构建伊布替尼耐药细胞株（MEC1 resistance，MR）：检测初始细胞系MEC1的半抑制浓度（IC_50_），将细胞系正常培养扩增后，以较低浓度的药物浓度进行培养；在药物诱导4 h后，去除药物，待细胞恢复24～72 h后再次进行药物培养；若细胞生长良好，则可以梯度增加药物浓度，使细胞尽快获得耐药性[12]。对不同耐药水平的细胞株进行冻存及耐药性的检测（可加入不同浓度的伊布替尼，以初始细胞系MEC1为对照，检测IC_50_或通过流式细胞术检测细胞凋亡水平），并在最后对诱导培养后的细胞系进行IC_50_的检测，耐药系数（RI）＝诱导细胞IC_50_/初始细胞IC_50_。

5. 慢病毒转染实验：铺设96孔板，设计感染复数（MOI）＝10、20、50、100四个浓度，加入病毒后感染72 h，于荧光显微镜下观察到MOI＝50时细胞转染效率>80％，且细胞状态较好，故选择此实验条件进行后续实验。96孔板每孔加入2×10^5^个细胞。加入MOI＝50时所需的病毒悬液及HiTransG P转染试剂（上海吉凯基因医学科技股份有限公司，中国上海），混匀后置培养箱培养24 h。之后补入新鲜培养液继续培养48 h，然后换液并重悬细胞，加入嘌呤霉素（赛默飞世尔科技有限公司，美国）筛选。筛选1周后，使用荧光显微镜或流式细胞术观察转染效率，直至转染效率>90％。使用qRT-PCR技术检测BIM mRNA表达情况，进行后续研究。

6. 小干扰RNA（siRNA）转染实验：前期实验确定siRNA浓度为100 nmol/L时细胞转染效率最佳且细胞状态良好，故选用此条件进行后续实验。在12孔板中每孔加入2×10^5^个/ml浓度细胞悬液1 ml。准备Lipofectamine 3000试剂（赛默飞世尔科技有限公司，美国），以每孔1 µl的量取出，加入至50 µl Opti-MEM溶液（赛默飞世尔科技有限公司，美国）中。在室温下避光孵育5 min。准备siRNA（上海吉玛制药技术有限公司，中国上海），加入DEPC水溶解。后使用Opti-MEM溶液分别稀释终浓度为100 nmol/L的病毒悬液。将孵育后的稀释siRNA悬液和孵育后的Lipofectamine 3000试剂混合，再次避光孵育20 min，制备siRNA-Lipofectamine 3000混合液。将混合液加入12孔板中，充分混匀。转染12 h后，可使用qRT-PCR技术验证自噬相关基因（ATG）5敲低效率，并收集细胞进行后续细胞功能实验。

7. CCK-8法绘制细胞增殖曲线：准备状态良好的细胞，分为对照组和加药组，设置0、24、48、72 h四个时间观察组，每组3个复孔。在96孔板接种细胞，加药组加入药物，对照组加培养液，每孔总体积100 µl。培养后按设置时间点加CCK-8试剂（Dojindo公司，日本），避光孵育2 h后，用酶标仪测细胞吸光度（*A*）。汇总*A*值，绘制细胞增殖曲线。

8. 细胞凋亡检测：准备状态较好的细胞，正常处理或shRNA、siRNA转染后接种于6孔板，每孔5×10^5^个细胞。加药组加药，对照组加培养液，总体积2 ml。充分混匀后放置在培养箱内培养24 h后，转至流式管，离心洗涤。加抗体（BD公司，美国）后避光孵育20 min，用流式细胞仪检测。

9. 免疫共沉淀技术（CoIP）：将细胞在RIPA缓冲液中裂解，并以12 000×*g*离心20 min。将裂解的样品与蛋白A/G琼脂糖珠在4 °C下孵育2 h，以阻止非特异性结合，期间保持旋转。随后，向样品中加入特异性抗体或相同种类的IgG（比例为1 µg抗体∶1 mg细胞蛋白），并在4 °C下孵育过夜。第2天再次加入20～80 µl蛋白A/G琼脂糖珠，继续孵育3 h。孵育后用RIPA缓冲液充分洗涤样品。最后，将沉淀的蛋白质在2×SDS样品缓冲液中煮沸洗脱，并进行SDS-PAGE分析。

10. RNA测序（RNA-seq）：本实验委托北京诺禾致源生物科技有限公司进行转录组测序。首先，提取细胞总RNA，后检测其完整性和总量。富集polyA尾mRNA并打断，以片段化mRNA为模板合成cDNA第1条链。降解RNA后合成第2条链，纯化双链cDNA并进行末端修复、加polyA尾、连接测序接头。筛选370～420 bp的cDNA进行PCR扩增并纯化，完成文库构建。使用llumina NovaSeq 6000测序。

11. 统计学处理：本实验应用SPSS和Graphpad prism软件进行作图和统计分析。使用独立样本*t*检验对两组变量间差异进行分析。使用方差分析（ANOVA）对两组以上的变量间差异进行分析。每次实验独立重复3次，*P*<0.05为差异有统计学意义。

## 结果

1. CLL在伊布替尼耐药时凋亡蛋白变化以促凋亡蛋白表达下降为主：对3例患者伊布替尼耐药前后的CD19^+^ B淋巴细胞进行RNA-seq高通量测序。以log_2_ Fold Change绝对值>0.5且*P*<0.05作为筛选条件对表达谱数据进行差异分析，结果显示伊布替尼敏感组和耐药组间有3 628个差异表达基因（DEG），耐药组1 077个基因高表达、2 551个基因低表达。DEG与BCL2家族的韦恩分析显示二者交集的基因共9个，其中多数为促凋亡蛋白（图1A）。对BCL2家族基因的差异倍数排序，提示伊布替尼耐药时BCL2家族基因表达变化以促凋亡基因表达下降为主，其中BH3-only蛋白BIM mRNA下降最为明显（降低87.92％，*P*<0.0001）（图1B）。为进一步验证伊布替尼耐药时BIM表达变化，我们在MEC1细胞株中使用小剂量浓度梯度诱导法，构建MR。通过RNA-seq技术检测分析MEC1细胞及MR中BCL2家族的表达变化（图1C）。与原代细胞结果相一致，MR中存在多种促凋亡基因的表达降低，其中BIM mRNA表达下降（图1D）。伊布替尼处理后，相较于MEC1细胞，MR中BIM的蛋白表达量明显下降（图1E）。此外，随着细胞耐药水平逐步升高，BIM mRNA表达逐步降低（*P*<0.05，图1F），提示BIM在CLL伊布替尼耐药中可能发挥重要作用。

**图1:**
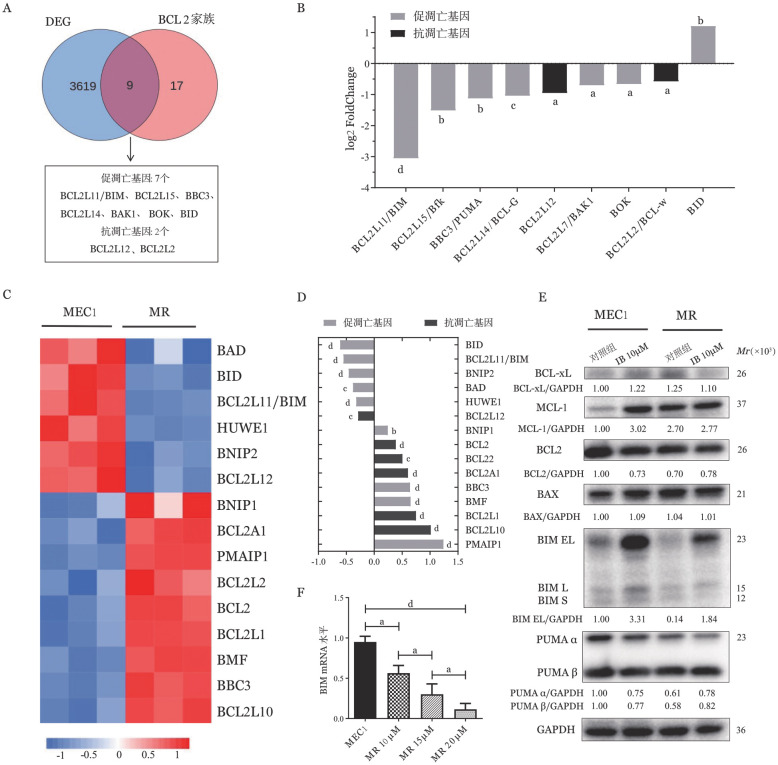
BCL2家族基因表达差异分析揭示BIM在慢性淋巴细胞白血病伊布替尼耐药中的关键作用 **A** 韦恩图筛选出DEG与BCL2家族之间存在交集的基因；**B** BCL2家族各基因在伊布替尼耐药前后的表达差异倍数；**C** 耐药细胞株（MR）和MEC1细胞中BCL2家族差异基因表达热图；**D** BCL2家族各基因在MR和MEC1细胞间的表达差异倍数；**E** MEC1及MR伊布替尼处理前后BIM蛋白的表达变化；**F** 诱导耐药前（MEC1）、诱导耐药至10 µmol/L（MR10）、15 µmol/L（MR15）、20 µmol/L（MR20）时BIM mRNA表达变化 **注** IB：伊布替尼；µM：µmol/L；ns为差异无统计学意义；^a^为*P*<0.05；^b^为*P*<0.01；^c^为*P*<0.001；^d^为*P*<0.0001

2. 伊布替尼药物作用依赖BIM表达，BIM低表达可诱导伊布替尼药物抗性：为阐明BIM在伊布替尼药物中的作用，我们首先检测伊布替尼敏感细胞株中细胞凋亡时BIM的变化情况。BIM同工型之一的BIM EL的表达最为丰富，因此我们通过检测BIM EL来反映BIM的表达变化。在伊布替尼敏感的MEC1细胞中，使用伊布替尼10 µmol/L、15 µmol/L分别作用24 h后发现BIM EL和PARP CF蛋白（PARP剪切产物，即Cleaved PARP）表达均增加且具有浓度依赖性（图2A）。进一步动态检测伊布替尼10 µmol/L作用不同时间的蛋白变化情况，结果显示细胞凋亡PARP CF蛋白表达随着BIM EL表达增加而增加，且具有时间依赖性（图2B）。相关性分析也显示，PARP CF的表达水平与BIM EL呈强相关性（*R*＝0.98，*P*<0.005，图2C）。以上结果均证明，伊布替尼的促凋亡作用依赖BIM介导。为进一步明确BIM低表达对CLL细胞生物学功能的影响，我们设计3条针对BIM的shRNA序列（shBIM#1、shBIM#2和shBIM#3）构建BIM敲低稳转细胞株（图2D、E）。

**图2:**
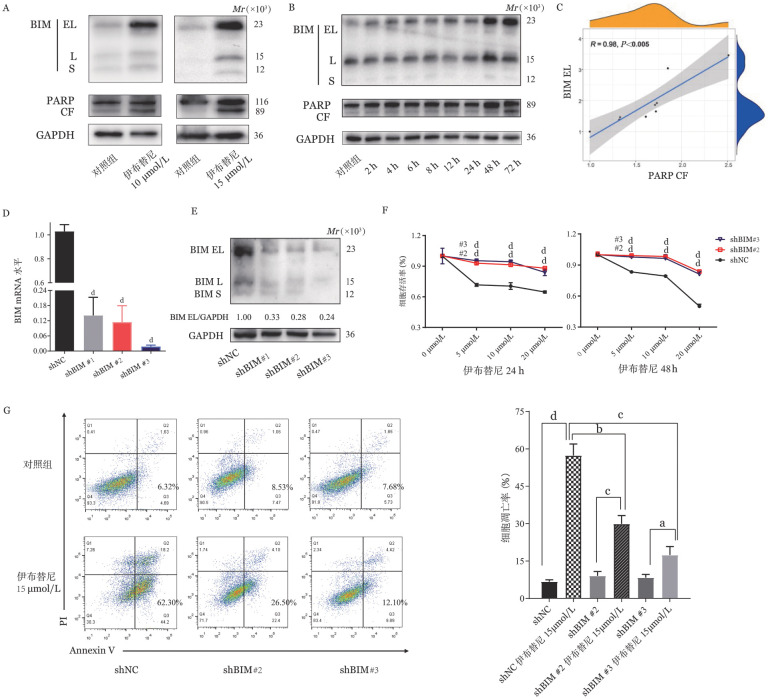
BIM参与伊布替尼诱导细胞凋亡及其低表达可诱导慢性淋巴细胞白血病伊布替尼耐药 **A** MEC1细胞中未加药物（对照组）、伊布替尼10 µmol/L、伊布替尼15 µmol/L作用24 h后的BIM、PARP及剪切产物PARP CF蛋白表达情况；**B** 在MEC1细胞中通过Western blot检测伊布替尼10 µmol/L作用不同时间点的BIM、PARP及剪切产物PARP CF蛋白表达情况；**C** PARP CF和BIM EL表达之间的相关性的可视化绘图；**D、E** 敲低组和对照组（shNC）BIM mRNA和蛋白表达情况；**F** 在shNC和敲低组中分别使用不同浓度伊布替尼处理细胞24 h、48 h后，通过CCK-8检测细胞活力；**G** 在shNC和敲低组中使用伊布替尼15 µmol/L处理细胞48 h后，通过流式细胞术检测细胞凋亡比例 **注** shNC：对照；^a^为*P*<0.05；^b^为*P*<0.01；^c^为*P*<0.001；^d^为*P*<0.0001

挑选其中敲低效率较高的两株（shBIM#2和shBIM#3）进行细胞功能实验。CCK-8结果显示，BIM敲低组在24 h或48 h细胞活力均明显高于对照组细胞，且差异具有统计学意义（*P*<0.05，图2F）。伊布替尼15 µmol/L作用24 h后，对照组（shNC）、shBIM#2和shBIM#3三组中发生凋亡的细胞分别占总细胞62.30％、26.50％和12.10％（图2G）。提示细胞对伊布替尼的敏感性依赖BIM的表达，下调BIM可使CLL细胞出现对伊布替尼的抗性。

3. 由BIM介导激活的保护性自噬可诱导CLL细胞产生伊布替尼耐药：RNA-seq结果显示，MR中ATG2、ATG3、ATG5、ATG9、ATG10等表达高于MEC1细胞（*P*<0.05，图3A）。使用透射电镜在MR内明显观察到具有双侧膜结构的自噬小体，提示存在自噬现象，而MEC1细胞中未见相关结构（图3B）。同时Western blot结果显示MR中自噬相关蛋白LC3 Ⅱ蛋白表达增加、自噬底物P62表达减少（图3C）。上述实验均证明，伊布替尼耐药时细胞自噬水平增高。在伊布替尼加药后不同时间点收集细胞，动态观察MR中LC3-Ⅱ和P62蛋白表达情况。如图3D显示，在加药后早期（2 h）LC3-Ⅱ蛋白表达便开始增高，12～24 h达到高峰；自噬底物P62蛋白从8 h起出现下降，12～24 h降低最为明显；以上提示耐药细胞在药物作用早期即出现自噬水平上升且自噬流完整。同样我们发现使用氯喹抑制MR的自噬后，肿瘤细胞增殖减少（图3E）、凋亡增加（图3F、G），提示MR通过上调自噬水平来逃避药物诱导的凋亡。为进一步探究BIM是否同时参与保护性自噬的调控，我们检测了BIM敲低细胞株中自噬相关蛋白LC3-Ⅱ的变化情况。和对照细胞shNC相比，shBIM#2和shBIM#3中BIM蛋白表达下降，而LC3-Ⅱ蛋白表达增加，提示BIM降低可介导自噬激活（图3H）。同时，CoIP-Western blot结果显示CLL细胞中BIM与Beclin-1之间并无相互结合关系（图3I），提示CLL中BIM可能通过拮抗其他关键的靶点蛋白抑制自噬。

**图3:**
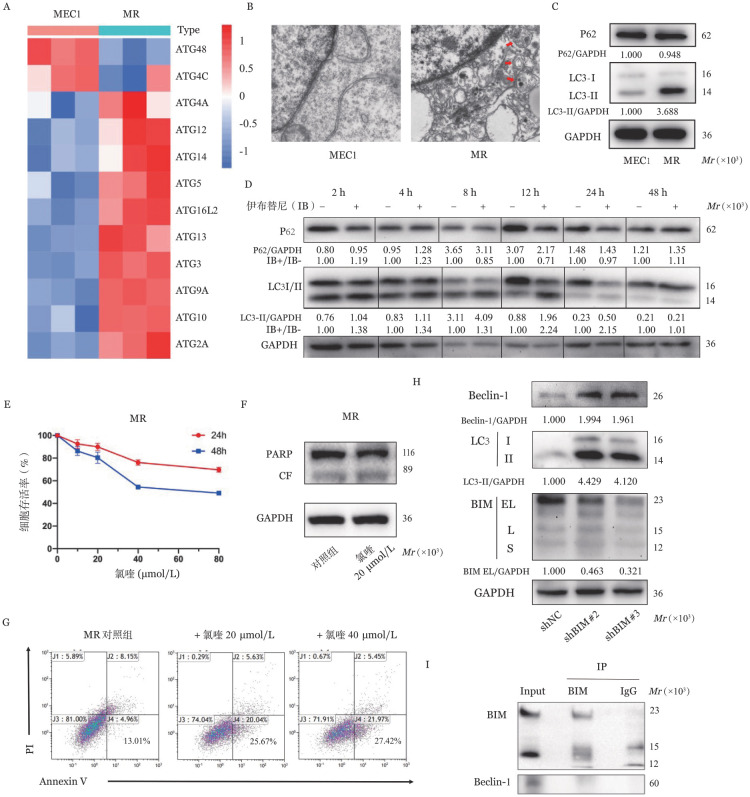
伊布替尼耐药CLL细胞中自噬水平上升与BIM表达降低相关 **A** MR和MEC1基因自噬相关基因表达热图；**B** 透射电镜下MEC1和MR细胞内自噬体等相关细胞结构（红色箭头指向自噬小体）；**C** 耐药前后自噬相关蛋白LC3-Ⅰ、LC3-Ⅱ和P62蛋白表达情况；**D** MR细胞中伊布替尼作用后不同时间点LC3-Ⅱ和P62蛋白表达情况；**E-G** MR细胞中不同浓度自噬抑制剂氯喹作用24 h和48 h后的细胞增殖、凋亡情况；**H** shBIM敲低组和对照组（shNC）中BIM、LC3及Beclin-1的蛋白表达情况；**I** 免疫共沉淀-蛋白质印迹法验证BIM和Beclin-1之间相互作用关系

## 讨论

本研究纳入了3例CLL患者，在接受伊布替尼治疗后2例部分缓解、1例完全缓解。后3例患者均发生了疾病进展，疾病的进展时间分别为服药后18、11、26个月，与既往报道[13]的伊布替尼疗效和耐药数据一致。

目前在CLL中导致伊布替尼耐药的主要原因被认为是BTK和PLCG2位点的突变。但其无法完全解释患者耐药。Woyach等[14]报道17％（6/35）的患者未发现BTK或PLCG2的突变。Ahn等[15]报道20％（2/10）的患者未发现耐药基因的突变。同时，本研究也未在诱导的MR中检测出BTK和PLCG2位点的突变，然而实验中MR依旧表现出对伊布替尼药物极低的敏感性。以上均提示除了单纯位点突变，伊布替尼的获得性耐药仍存在其他机制。

细胞凋亡和增殖的失衡是恶性肿瘤发生的关键原因。线粒体途径凋亡由BCL2家族调控，该家族包括促凋亡和抗凋亡蛋白。促凋亡蛋白分为多结构域（如BAX、BAK）和单结构域（BH3-only，如BIM、PUMA等）两类；抗凋亡蛋白包括BCL2、BCL-xL等。当凋亡信号激活时，BAX和BAK在线粒体形成跨膜孔，从而改变线粒体膜通透性，释放线粒体内凋亡分子，而抗凋亡蛋白通过结合BAX和BAK抑制凋亡。BH3-only蛋白通过直接或间接作用激活凋亡[16]。促凋亡与抗凋亡蛋白协同作用，共同调节细胞凋亡平衡。

凋亡蛋白表达失衡是血液系统恶性肿瘤的重要特征。与正常B淋巴细胞相比，CLL细胞中BCL2、MCL-1和BCL-xL表达增加，BAX表达减少[17]。BH3-only蛋白BIM和NOXA表达可能会代偿性增加[18]，其主要通过竞争抗凋亡蛋白发挥作用而非直接激活凋亡。肿瘤药物的促凋亡作用往往依赖于BCL2家族调节，凋亡蛋白表达的失衡与耐药密切相关，如急性髓细胞白血病（AML）中ABT-199耐药与MCL-1和BCL-xL表达增加有关[19]，小细胞肺癌中顺铂耐药与BCL2升高及BIM下降有关[20]。本研究伊布替尼耐药主要表现为促凋亡蛋白BIM下降，而抗凋亡蛋白上升不明显，一方面由于个体异质性，另一方面MCL-1等抗凋亡蛋白存在选择性剪切，其长、短片段（MCL-1 L和MCL-1 S）对凋亡作用不同，伊布替尼耐药时主要表现为MCL-1 L/S比例变化，而其总表达量可能不变[21]。

BIM是BH3-only蛋白中重要的促凋亡蛋白，由于mRNA的选择性剪切，BIM共有19种不同的同工型，其中主要为BIM S、BIM L和BIM EL三种，发挥不同的促凋亡活性[22]。BIM EL是人体组织主要的同工型，也是本研究中观察BIM表达变化的主要对象。BIM的功能异常已被证明是多种肿瘤药物耐药的重要原因[23]–[25]。

在EGFR突变的非小细胞肺癌中，EGFR激酶结构域突变激活MEK/ERK通路，抑制BIM表达，从而导致肿瘤细胞对厄洛替尼耐药[26]。在BCR^-^ABL^+^白血病小鼠中，突变细胞通过IL-3旁分泌激活非突变细胞MEK/ERK和JAK2/STAT5通路，下调BIM表达，从而产生对伊马替尼和达沙替尼的抗性[27]。在多发性骨髓瘤中，BIM下调同样促使细胞对硼替佐米的耐药[28]。本研究原代伊布替尼耐药细胞、耐药株MR中BIM表达均明显下降。少部分BCL2家族基因表达趋势不同（BCL2 L2、BID），可能由于原代细胞与细胞系之间存在差异。此外，随着CLL细胞系中伊布替尼耐药性的增强，BIM的表达逐步降低，提示BIM低表达与伊布替尼耐药密切相关。进一步实验发现，敲低BIM能够显著削弱伊布替尼的抗增殖和促凋亡作用，表明BIM在药物杀伤作用中至关重要。目前伊布替尼耐药时BIM下调的机制尚未完全明确，可能是FOXO3a活性下降导致，后者受PI3K/AKT通路旁路激活影响，抑制FOXO3a转录功能，从而下调BIM表达[29]。

BIM不仅调控细胞凋亡，还参与细胞自噬。自噬是一个复杂动态过程，由ATG严格调控。LC3是自噬的关键蛋白，其活性形式LC3-Ⅱ与自噬体数量呈正相关；ATG5是自噬体膜延伸的关键基因[30]。前期研究[9]发现，CLL患者自噬水平升高且ATG5 mRNA高表达。本实验通过检测LC3-Ⅱ及ATG5表达来探讨自噬在伊布替尼耐药中的作用，结果显示耐药细胞自噬水平显著升高。使用自噬抑制剂能增加细胞凋亡，表明自噬作为一种保护机制促进耐药。BIM可以与重要的自噬相关蛋白Beclin-1的BH3结构域结合，将Beclin-1从内质网错位至微管，从而抑制细胞自噬激活[16]。然而本研究CoIP-Western blot结果显示CLL细胞中BIM与Beclin-1无相互结合，提示CLL中BIM未通过Beclin-1/LC3经典通路激活自噬，而可能通过拮抗其他关键的靶点蛋白从而抑制自噬。本研究存在一定的局限性，研究样本量偏小，在后续的研究中，我们将通过扩大临床样本规模，充分验证伊布替尼耐药与BIM低表达的相关性。

## References

[1] O'Donnell A, Pepper C, Mitchell S (2023). NF-kB and the CLL microenvironment[J]. Front Oncol.

[2] Rozkiewicz D, Hermanowicz JM, Kwiatkowska I (2023). Bruton's tyrosine kinase inhibitors (BTKIs): review of preclinical studies and evaluation of clinical trials[J]. Molecules.

[3] Reyes A, Siddiqi T (2023). Targeting BCL2 pathways in CLL: a story of resistance and ingenuity[J]. Cancer Drug Resist.

[4] Wang E, Mi X, Thompson MC (2022). Mechanisms of resistance to noncovalent Bruton's tyrosine kinase inhibitors[J]. N Engl J Med.

[5] 陈 骢, 郝 健淇, 彭 皓宁 (2023). BCL-2家族凋亡调控作用及其介导的抗肿瘤药物治疗后耐药的研究进展[J]. 中国胸心血管外科临床杂志.

[6] Avsec D, Škrlj Miklavčič M, Burnik T (2022). Inhibition of p38 MAPK or immunoproteasome overcomes resistance of chronic lymphocytic leukemia cells to Bcl-2 antagonist venetoclax[J]. Cell Death Dis.

[7] Qiu Y, Li Y, Chai M (2023). The GSK3β/Mcl-1 axis is regulated by both FLT3-ITD and Axl and determines the apoptosis induction abilities of FLT3-ITD inhibitors[J]. Cell Death Discov.

[8] Debnath J, Gammoh N, Ryan KM (2023). Autophagy and autophagy-related pathways in cancer[J]. Nat Rev Mol Cell Biol.

[9] Kopeina GS, Zhivotovsky B (2023). The New face of autophagy in chronic lymphocytic leukemia[J]. Cancers (Basel).

[10] Hu X, Wen L, Li X (2023). Relationship between autophagy and drug resistance in tumors[J]. Mini Rev Med Chem.

[11] Hu Y, Wen Z, Liu S (2020). Ibrutinib suppresses intracellular mycobacterium tuberculosis growth by inducing macrophage autophagy[J]. J Infect.

[12] Xu Z, Pan B, Miao Y (2023). Prognostic value and therapeutic targeting of XPO1 in chronic lymphocytic leukemia[J]. Clin Exp Med.

[13] Burger JA, Tedeschi A, Barr PM (2015). Ibrutinib as initial therapy for patients with chronic lymphocytic leukemia[J]. N Engl J Med.

[14] Woyach JA, Ruppert AS, Guinn D (2017). BTK(C481S)-mediated resistance to ibrutinib in chronic lymphocytic leukemia[J]. J Clin Oncol.

[15] Ahn IE, Tian X, Ipe D (2021). Prediction of outcome in patients with chronic lymphocytic leukemia treated with ibrutinib: development and validation of a four-factor prognostic model[J]. J Clin Oncol.

[16] Saha A, Saleem S, Paidi RK (2021). BH3-only proteins Puma and Beclin1 regulate autophagic death in neurons in response to Amyloid-β[J]. Cell Death Discov.

[17] Mohammadlou M, Abdollahi M, Hemati M (2021). Apoptotic effect of berberine via Bcl-2, ROR1, and mir-21 in patients with B-chronic lymphocytic leukemia[J]. Phytother Res.

[18] Kubiak AB, Ziółkowska EI, Korycka-Wołowiec AB (2022). The influence of venetoclax, used alone or in combination with cladribine (2-CdA), on CLL cells apoptosis in vitro: Preliminary results[J]. Adv Clin Exp Med.

[19] Glytsou C, Chen X, Zacharioudakis E (2023). Mitophagy promotes resistance to BH3 mimetics in acute myeloid leukemia[J]. Cancer Discov.

[20] Gay CM, Stewart CA, Park EM (2021). Patterns of transcription factor programs and immune pathway activation define four major subtypes of SCLC with distinct therapeutic vulnerabilities[J]. Cancer Cell.

[21] Li Y, Gao X, Wei C (2020). Modification of Mcl-1 alternative splicing induces apoptosis and suppresses tumor proliferation in gastric cancer[J]. Aging (Albany NY).

[22] Khandia R, Ali Khan A, Alexiou A (2022). Codon usage analysis of pro-apoptotic bim gene isoforms[J]. J Alzheimers Dis.

[23] Scherr M, Kirchhoff H, Battmer K (2019). Optimized induction of mitochondrial apoptosis for chemotherapy-free treatment of BCR-ABL+acute lymphoblastic leukemia[J]. Leukemia.

[24] Tanaka K, Yu HA, Yang S (2021). Targeting Aurora B kinase prevents and overcomes resistance to EGFR inhibitors in lung cancer by enhancing BIM- and PUMA-mediated apoptosis[J]. Cancer Cell.

[25] Tu YS, He J, Liu H (2017). The imipridone ONC201 induces apoptosis and overcomes chemotherapy resistance by up-regulation of bim in multiple myeloma[J]. Neoplasia.

[26] Kitai H, Choi PH, Yang YC (2024). Combined inhibition of KRAS(G12C) and mTORC1 kinase is synergistic in non-small cell lung cancer[J]. Nat Commun.

[27] Yu M, Nah G, Krishnan V (2025). The BIM deletion polymorphism potentiates the survival of leukemia stem and progenitor cells and impairs response to targeted therapies[J]. Leukemia.

[28] Huang L, Wang Y, Bai J (2020). Blockade of HSP70 by VER-155008 synergistically enhances bortezomib-induced cytotoxicity in multiple myeloma[J]. Cell Stress Chaperones.

[29] Kapoor I, Li Y, Sharma A (2019). Resistance to BTK inhibition by ibrutinib can be overcome by preventing FOXO3a nuclear export and PI3K/AKT activation in B-cell lymphoid malignancies[J]. Cell Death Dis.

[30] Feng X, Zhang H, Meng L (2021). Hypoxia-induced acetylation of PAK1 enhances autophagy and promotes brain tumorigenesis via phosphorylating ATG5[J]. Autophagy.

